# Association of Night Sleep Duration and Ideal Cardiovascular Health in Rural China: The Henan Rural Cohort Study

**DOI:** 10.3389/fpubh.2020.606458

**Published:** 2021-01-11

**Authors:** Xueyan Wu, Xiaotian Liu, Wei Liao, Ning Kang, Shengxiang Sang, Tanko Abdulai, Zhihan Zhai, Chongjian Wang, Xiaoqiong Wang, Yuqian Li

**Affiliations:** ^1^Department of Epidemiology and Biostatistics, College of Public Health, Zhengzhou University, Zhengzhou, China; ^2^Department of Economics, School of Business, Zhengzhou University, Zhengzhou, China; ^3^Department of Clinical Pharmacology, School of Pharmaceutical Science, Zhengzhou University, Zhengzhou, China

**Keywords:** ideal cardiovascular health, health behaviors, health factors, night sleep duration, rural population

## Abstract

**Introduction:** We aimed to explore the association between night sleep duration and ideal cardiovascular health (ICH) among Chinese rural population.

**Methods:** In all, 35,094 participants were included from the Henan Rural Cohort study. Information on sleep was collected using the Pittsburgh Sleep Quality Index. The ICH scores were evaluated. The associations between night sleep duration and ICH were examined using both linear regression and logistic regression models.

**Results:** The mean night sleep duration for all participants was 7.75 ± 1.28 h. Compared with those with night sleep duration of 7 to <9 h by using linear regression model, a significant decrease in ICH scores was observed for participants with shorter [−0.077 (−0.131, −0.024)] and longer [−0.079 (−0.121, −0.036)] night sleep duration. Compared with 7 to <9 h, longer sleep duration [0.919 (0.851, 0.992)] were associated with decreased odds of ideal CVH.

**Conclusions:** Shorter and longer night sleep duration are negatively associated with ICH among rural population. This suggests that it may be beneficial to include night sleep duration assessment in cardiovascular risk screening.

## Introduction

Cardiovascular disease (CVD) is now the leading cause of premature mortality and disability both worldwide (1, 2) and in China (3). There are the heavy social burdens of disease due to CVD in China (4). In 2010, ideal cardiovascular health (ICH) was defined as the simultaneous presence of four ideal health behaviors including ideal smoking status, ideal body mass index (BMI), ideal physical activity, and ideal diet and four ideal health factors including ideal smoking status, ideal total cholesterol (TC), ideal blood pressure (BP), and ideal fasting plasma glucose (FPG) in the absence of CVD history by the American Heart Association (AHA) (5). Prospective studies consistently indicated that individuals with higher number of ICH metrics have lower risks of CVD events, hypertension, type 2 diabetes mellitus, cancer and all-cause mortality (6–11).

In the past few years, several studies have evaluated the relationship between night sleep duration and the occurrence of hypertension (12), diabetes (13), CVD (14–18). However, most previous studies considered single cardiovascular risk factors. A National Health and Nutrition Examination Survey among US adults, reported that both shorter and longer sleep duration were associated with decreased odds ratio of ICH and lower mean cardiovascular health scores (19). However, for a population with limited resources, such as the rural population, limited work has assessed the association between night sleep duration and cardiovascular health. Hence, we performed the current study based on data from the Henan Rural Cohort. This study aimed to explore the association between sleep duration and cardiovascular health in rural individuals.

## Methods

### Study Design and Participants

The Henan Rural Cohort, was established in Henan Province, China during 2015–2017. A total of 39,259 people were included in the cohort study with a response rate of 93.7%. The details of this cohort have been described elsewhere (20). In current study, 39,223 participants with complete information on sleep information were included. Then, the participants were further excluded if they: (1) were diagnosed with coronary heart disease (*n* = 1,732); (2) were diagnosed with stroke (*n* = 2,639); (3) missed the necessary information in the present study (*n* = 99). Finally, 35,049 adults were included in the study.

Written informed consent was obtained from all participants. The study was approved by the “Zhengzhou University Life Science Ethics Committee” [Ethics approval code: [2015] MEC (S128)].

### Data Collection

Data on participants' demographic characteristics, lifestyles, behaviors, dietary patterns (FFQ), individual history of diseases and medication use were collected using standard questionnaire through face to face interviews by well-trained research staff. Weight and height were measured twice in light clothing with shoes off and recorded to the nearest 0.1 kg and 0.1 cm, respectively, and we calculated the average of the two measures. Body mass index (BMI) was computed as body weight (kg) divided by height square (m^2^) based on the measurement. Blood pressure was measured three times by electronic sphygmomanometer (Omron HEM-7071A, Japan) in the right arm in a sitting position after at least 5 min rest. There were 30 s intervals between the three measurements. Venous blood samples were collected from subjects after an overnight fast of at least 8 h and stored in −80°C cryogenic refrigerator before analysis. The fasting blood glucose (FBG) was analyzed via glucose oxidative method (GOD-PAP) by ROCHE Cobas C501 automatic biochemical analyzer. Total cholesterol was measured by Roche Cobas C501 automatic biochemical analyzer.

### Ideal Cardiovascular Health Scores

We used the AHA definitions of ICH (5). Each ICH metric was categorized as ideal and non-ideal according to the following criteria: ideal TC, TC < 5.18 mmol/L untreated; ideal FPG, FPG < 5.6 mmol/L untreated; ideal BP, SBP <120/DBP < 80 mm Hg untreated; ideal smoking status, never a smoker; ideal physical activity, physical activity ≥150 min/week of moderate intensity or ≥75 min/week of vigorous intensity or ≥150 min/week of moderate-vigorous intensity combination; ideal BMI, BMI < 25 kg/m^2^; ideal diet, ≥ 4 components. In addition, ICH was defined according to the American Heart Association's 2020 Strategic Impact Goals as follows: the simultaneous presence of 4 ideal health behaviors (ideal smoking status, ideal BMI, ideal PA, and ideal diet) and 4 ideal health factors (ideal smoking status, ideal TC, ideal BP, and ideal FPG) in the absence of a history of CVD. The healthy diet score was made some adaptations as appropriate. Healthy diet score was calculated by adding the number of diet components, including fruits and vegetables ≥500 g/d; fish ≥200 g/week; soybean products ≥125 g/d; red meat < 75 g/d; drinking tea. Ideal diet was defined as healthy diet score ≥4 components (21).

We calculated the ICH score by summing up the number of ideal metrics for each participant, ranging from 0 to 7, and participants were classified as having ideal CVH (≥5) and non-ideal CVH (0~4) (5). Ideal health behaviors (IHB) scores and ideal health factors (IHF) scores were calculated by summing the total number of IHB metrics and IHF metrics, respectively, both ranging from 0 to 4.

### Night Sleep Duration

Night sleep duration was evaluated by using the Pittsburgh Sleep Quality Index (PSQI) (22). Information on sleep was taken from answers to the following questions: (1) “What time have you usually gone to bed?” (bedtime), (2) “How long (in minutes) has it taken you to go to sleep each night during the past month?” (sleep latency), and (3) “What time have you usually gotten up in the morning?” (getting up time). Furthermore, we calculate the interval between bedtime and getting up time as night sleep duration. Night sleep duration was categorized as <6 h (shorter night sleep duration), 6 to <7 h, 7 to <9 h (reference), and ≥9 h (longer night sleep duration) (19).

### Statistical Analysis

Multivariable linear regression model was conducted to examine the association between night sleep duration and ICH scores. Beside the linear regression model, logistic regression model was used to further examine the association between night sleep duration and ideal CVH (ICH scores ≥5). A range of potential confounders were adjusted, including age (<40, 40–60 or ≥60 years), sex (men or women), educational level (primary school or illiteracy, junior high school, or high school or above), income (<500, 500–1,000 or ≥1,000 RMB per month) and drinking (no drinking or current drinking). Results were expressed as increased ICH scores and 95% confidence intervals (95%CIs) or odds ratio of ideal CVH associated with night sleep duration. The potential modification effects of sex, age, education level, income and drinking were examined by adding an interaction term into the adjusted model. All statistical analyses were performed by STATA 15 for Windows and R version 3.6.3.

## Results

### Characteristics of the Participants

The characteristics of the study participants by ICH groups are presented in Table 1. Of all 35,049 participants, 21,353 (60.92%) were women and the mean age was 54.74 ± 12.28 years. A total of 13,005 participants (37.11%) showed Ideal CVH (ICH scores ≥5) and nearly half were classified as intermediate overall CVH. The mean night sleep duration for all participants was 7.75 ± 1.28 h. Lower mean age and BMI, higher income, higher proportion of women, and married/cohabiting were observed among those with ideal ICH compared to non-Ideal ICH participants. Participants with ideal CVH tended to be non-smokers and non-drinkers. The prevalence of those who slept 7 to <9 h was 67.21%, and the prevalence of shorter sleep (<6 h) was 6.16% and longer sleep (≥9 h) was 10.44%. In the different ICH groups, the proportion of those who slept 7 to <9 h with ideal CVH was significantly higher than those with non-ideal CVH, while participants who were shorter sleep or longer sleep with ideal CVH were lower than those with non-ideal CVH.

**Table 1:** Characteristics of the participants.

**Variables**	**Total**	**Ideal CVH**	**Non-ideal CVH**	***p***
	**(*n* = 35,049)**	**(*n* = 13,005)^a^**	**(*n* = 22,044)^b^**	
**Age (years, mean ± SD)**	54.74 ± 12.28	51.25 ± 12.98	56.80 ± 11.351	<0.001
**Sex**				<0.001
Men	13,696 (39.08)	3,404 (26.17)	10,292 (46.69)	
Women	21,353 (60.92)	9,601 (73.83)	11,752 (53.31)	
**Marital status**				0.018
Married/cohabiting	31,611 (90.19)	11,793 (90.68)	19,818 (89.90)	
Unmarried/divorced/widowed	3,438 (9.81)	1,212 (9.32)	2,226 (10.10)	
**Education level**				<0.001
Primary school or illiteracy	15,128 (43.16)	5,227 (40.19)	9,901 (44.91)	
Junior high school	14,304 (40.81)	5,573 (42.85)	8,731 (39.61)	
High school or above	5,617 (16.03)	2,205 (16.96)	3,412 (15.48)	
**Income**				<0.001
<500	12,169 (34.72)	4,336 (33.34)	7,833 (35.53)	
500	11,623 (33.16)	4,260 (32.76)	7,363 (33.40)	
1,000	11,257 (32.12)	4,409 (33.90)	8,648 (31.07)	
**Smoking**				<0.001
No smoking	25,630 (73.13)	11,380 (87.50)	14,250 (64.64)	
Current smoking	9,419 (26.87)	1,625 (12.50)	7,794 (35.36)	
**Drinking**				<0.001
No drinking	27,078 (77.26)	11,203 (86.14)	15,875 (72.02)	
Current drinking	7,971 (22.74)	1,802 (13.86)	6,169 (27.98)	
**BMI (kg/m2 mean ± SD)**	24.79 ± 3.56	22.84 ± 2.76	25.94 ± 3.48	<0.001
**Night sleep duration, h**				<0.001
<6	2,160 (6.16)	738 (5.67)	1,422 (6.45)	
6 to <7	5,671 (16.18)	2,103 (16.17)	3,568 (16.19)	
7 to <9	23,558 (67.21)	8,913 (68.54)	14,645 (66.44)	
≥9	3,660 (10.44)	1,251 (9.62)	2,409 (10.93)	
**Mean night sleep duration, h**	7.75 ± 1.28	7.75 ± 1.24	7.76 ± 1.30	0.500

SD, standard deviation; Income, per capita monthly income, RMB per month; BMI, body mass index;

a ICH scores ≥5;

b *ICH scores, 0~4*.

### Associations Between Night Sleep Duration and ICH

**Figure 1:**
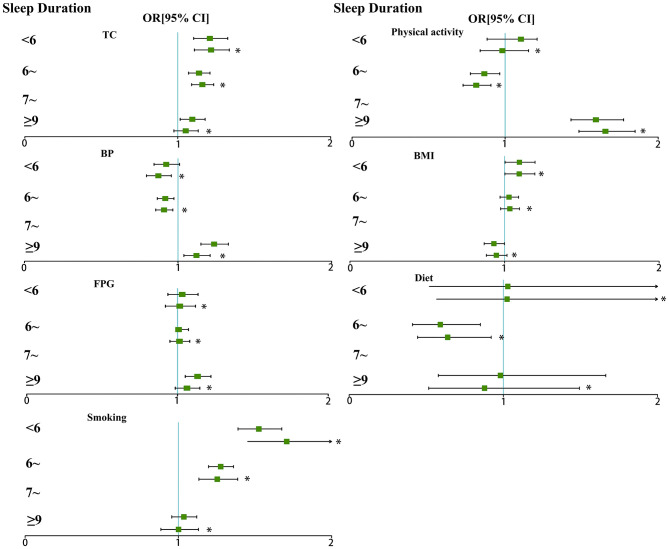
Odds ratio of ICH metrics (and 95%CI) associated with night sleep duration. *Potential confounders were adjusted, including age, sex, educational level, income and drinking.

Differences were significant in all ICH metrics when stratified by the reported night sleep duration (Table 2). The results of multivariable logistic regression models showed the odds ratios (and 95%CIs) of each ICH metrics which was associated with night sleep duration scores in Figure 1. After adjusting for potential confounders, shorter night sleep duration was strongly related with total cholesterol, BMI and smoking. A strong association between longer night sleep duration and ICH metrics was observed in physical activity and blood pressure. In the linear regression models, we found a consistent association between shorter and long night sleep duration and decreased ICH scores both in the unadjusted and adjusted models. After adjusting for potential confounders, a significant decrease was observed in ICH scores for participants with shorter [−0.077 (−0.131, −0.024)] and longer [−0.079 (−0.121, −0.036)] night sleep duration, compared with those with night sleep duration of 7 to <9 h. In the multivariable logistic regression models, compared to the participants who slept 7 to <9 h, OR (95%CI) for ideal CVH among those with shorter night sleep duration was 0.853 (0.777, 0.936) in the unadjusted model and the effect was slightly attenuated with an adjusted OR (95%CI) of 0.929 (0.844, 1.023). An association between longer night sleep duration and decreased odds of ideal CVH [unadjusted 0.853 (0.793, 0.918) and adjusted 0.919 (0.851, 0.992)] was also observed (Table 3). When examining the IHB scores and IHF scores as a continuous variable, the decreased IHB scores was associated with shorter night sleep duration [−0.063 (−0.091, −0.035)] and longer night sleep duration [−0.030 (−0.052, −0.008)] in the adjusted model, and similar results were observed that the decreased IHF scores increased associated with shorter night sleep duration and longer night sleep duration were −0.056 (−0.098, 0.014) and −0.048 (−0.081, −0.015) after adjusting (Supplementary Table 1).

**Table 2:** Prevalence of the ICH metrics by night sleep duration.

**ICH metrics**	**Total, n (%)**	**Night sleep duration, n (%)**				***p***
		**<6**	**6 to <7**	**7 to <9**	**≥9**	
**TC**						<0.001
Ideal	23,779 (67.85)	1,394 (64.54)	3,738 (65.91)	16,202 (68.77)	2,445 (66.80)	
Non-ideal	11,270 (32.15)	766 (35.46)	1,933 (34.09)	7,356 (31.23)	1,215 (33.20)	
**BP**						<0.001
Ideal	14,019 (40.00)	907 (41.99)	2,390 (42.14)	9,437 (40.06)	1,285 (35.11)	
Non-ideal	21,030 (60.00)	1,253 (58.01)	3,281 (57.86)	14,121 (59.94)	2,375 (64.89)	
**FPG**						0.013
Ideal	24,931 (71.13)	1,530 (70.83)	4,045 (71.33)	16,836 (71.47)	2,520 (68.85)	
Non-ideal	10,118 (28.87)	630 (29.17)	1,626 (28.67)	6,722 (28.53)	1,140 (31.15)	
**Smoking**						<0.001
Ideal	25,630 (73.13)	1,420 (65.74)	3,949 (69.63)	17,558 (74.53)	2,703 (73.85)	
Non-ideal	9,419 (26.87)	740 (34.26)	1,722 (30.37)	6,000 (25.47)	957 (26.15)	
**physical activity**						<0.001
Ideal	32,031 (91.39)	1,975 (91.44)	5,259 (92.73)	21,599 (91.68)	3,198 (87.38)	
Non-ideal	3,018 (8.61)	185 (8.56)	412 (7.27)	1,959 (8.32)	462 (12.62)	
**BMI**						0.017
Ideal	19,094 (54.48)	1,129 (52.27)	3,053 (53.84)	12,851 (54.55)	2,061 (56.31)	
Non-ideal	15,955 (45.52)	1,031 (47.73)	2,618 (46.16)	10,707 (45.45)	1,599 (43.69)	
**Diet**						0.034
Ideal	167 (0.48)	9 (0.42)	41 (0.72)	101 (0.43)	16 (0.44)	
Non-ideal	34,882 (99.52)	2,151 (99.58)	5,630 (99.28)	23,457 (99.57)	3,644 (99.56)	

*ICH, Ideal cardiovascular health; TC, total cholesterol; BP, blood pressure; FPG, fasting plasma glucose; BMI, body mass index (kg/m^2^)*.

*Ideal diet was defined as healthy diet score ≥4 components*.

**Table 3:** Association between night sleep duration categories and increased ICH scores and odds ratio of ideal CVH.

**Night sleep duration**	**Increased ICH scores (95%CI)**				**OR (95%CI)**			
	**Model 1^a^**	***p***	**Model 2^b^**	***p***	**Model 1^a^**	***p***	**Model 2^b^**	***p***
<6	−0.143 (−0.200, −0.086)	<0.001	−0.077 (−0.131, −0.024)	0.005	0.853 (0.777,0.936)	0.001	0.929 (0.844, 1.023)	0.133
6 to <7	−0.052 (−0.089, 0.014)	0.007	−0.022 (−0.057, 0.013)	0.219	0.968 (0.912, 1.028)	0.295	1.004 (0.943, 1.069)	0.892
7 to <9	0 [Reference]		0 [Reference]		1 [Reference]		1 [Reference]	
≥9	−0.128 (−0.172, −0.083)	<0.001	−0.079 (−0.121, −0.036)	<0.001	0.853 (0.793, 0.918)	<0.001	0.919 (0.851, 0.992)	0.031

*Ideal CVH, ICH scores≥5; ICH, Ideal cardiovascular health; Cl, confidence interval; OR, odds ratio*.

a *Model 1: Unadjusted*.

b *Model 2: Adjusted for demographic factors of age, sex, education level, income, and drinking*.

### Interaction Analyses of Night Sleep Duration and ICH

In Table 4, it is shown that the association between night sleep duration and cardiovascular health was modified by age, sex, income, educational level, and drinking. Stronger associations between shorter night sleep duration and increased ICH scores were present among men participants, who were 40 to <60 years, non-drinkers and with high education level. Conversely, stronger associations between longer night sleep duration and increased ICH scores were present among women who were aged ≥60 years, low income, with low education level, and non-drinker.

**Table 4:** Results of stratified analyses for the association between night sleep duration and ICH scores.

**Interaction term**	**Night sleep duration [increased ICH scores (95%CI)]**			
	**<6**	**6 to <7**	**7 to <9**	**≥9**
**Gender**				
Men	−0.118 (−0.204, −0.033)	−0.025 (−0.082, 0.033)	[Reference]	−0.073 (−0.114, −0.001)
Women	−0.020 (−0.087, 0.047)	0.002 (−0.042, 0.045)	[Reference]	−0.087 (−0.138, −0.036)
**Age**				
<40	−0.184 (−0.381, 0.012)	−0.147 (−0.255, −0.040)	[Reference]	−0.059 (−0.183, 0.064)
40–60	−0.094 (−0.168, −0.020)	−0.020 (−0.069, 0.030)	[Reference]	−0.077 (−0.148, −0.005)
≥60	0.004 (−0.082, 0.090)	0.007 (−0.051, 0.064)	[Reference]	−0.115 (−0.174, −0.057)
**Income**				
<500	−0.046 (−0.138, 0.046)	−0.022 (−0.083, 0.039)	[Reference]	−0.124 (−0.191, −0.058)
500	−0.137 (−0.232, −0.042)	−0.017 (−0.078, 0.044)	[Reference]	−0.081 (−0.156, −0.005)
1,000	−0.064 (−0.158, 0.031)	−0.056 (−0.119, 0.007)	[Reference]	−0.043 (−0.127, 0.040)
**Education level**				
Primary school or illiteracy	−0.017 (−0.072, 0.039)	−0.016 (−0.107, 0.076)	[Reference]	−0.098 (−0.170, −0.026)
Junior high school	−0.100 (−0.185, −0.015)	−0.073 (−0.128, −0.018)	[Reference]	−0.133 (−0.208, −0.058)
High school or above	−0.249 (−0.393, −0.105)	−0.031 (−0.119, 0.056)	[Reference]	0.052 (−0.081, 0.185)
**Drinking**				
No drinking	−0.068 (−0.131, −0.006)	−0.027 (−0.067, 0.013)	[Reference]	−0.094 (−0.141, −0.047)
Current drinking	−0.096 (−0.204, 0.012)	−0.039 (−0.115, 0.038)	[Reference]	−0.048 (−0.147, 0.051)

*Income, per capita monthly income; RMB per month; ICH, Ideal cardiovascular health*.

## Discussion

To the best of our knowledge, this is the first study to examine the effect of night sleep duration on ICH among rural adults. The proportion of those who slept 7 to <9 h with ideal CVH were higher than those with non-ideal CVH, while participants who with shorter sleep or longer sleep with ideal CVH were lower than those with non-ideal CVH. There was a strong correlation between shorter night sleep duration and ICH metrics including total cholesterol, BMI and smoking, and a strong association between longer sleep and ICH metrics was observed in physical activity and blood pressure. Shorter (<6 h) and longer night (≥9 h) sleep duration were significantly associated with decreasing ICH scores, IHB scores and IHF scores, and longer sleep duration were associated with decreased odds of ideal CVH.

Currently, evidence for the adverse effect of sleep duration on ICH is limited in China or elsewhere in the world. In a National Health and Nutrition Examination Survey among US adults, shorter and longer sleep duration were associated with decreased odds ratio of ICH and lower mean cardiovascular health scores (19). The MORGEN study found that those individuals who slept 6 h or less had a 15% higher risk of CVD incidence and a 23% higher risk of CHD incidence compared with people who slept 7–8 h (23). Some studies found a U-shaped association between sleep duration and CVD (24–26). Similar results were found in our study, and our results completed the association between night sleep duration and ICH among Chinese rural population, and the correlation intensity between shorter and longer duration of night sleep and ICH of the rural population is stronger than that of the developed countries. However, other studies have shown that long sleep duration has a protective effect on CVD or has non-significant associations (27). The effect estimates are not comparable due to differences in exposure and outcome assessments. Therefore, more studies are needed in the future to provide more solid and robust evidence for the relationship between night sleep duration and ICH.

The biological mechanism for sleep and CVH likely differs from the association between sleep duration and ICH previously described, which may have multiple pathways. Night sleep duration is independently associated with several ICH metrics. In our findings, after adjusting for potential confounders, there was a strong correlation between shorter night sleep duration and ICH metrics including total cholesterol, BMI and smoking, while the association between longer sleep and ICH metrics was observed in physical activity and blood pressure. Prior literature has demonstrated that sleep duration was independently associated with several CVH metrics, such as BMI (28) hypertension (26) and hyperlipidemia (15). Abnormal lipid profiles have been reported as a possible mechanism in individuals who sleep for a long duration (29). Compared to a long sleep duration, a short sleep duration is associated with several correlates. Studies have found that short sleep shows better morbidity prediction (30). We further found that stronger effects of shorter night sleep duration and increased ICH scores were present among men participants, who were 40 to <60 years, non-drinkers and with high education level and the associations between longer night sleep duration and increased ICH scores were present among women who were aged ≥60 years, low income, with low education level, and non-drinker. In the USA data, long sleep duration was associated with stroke, especially in older people (31). The elderly are prone to sleep disorders, and their sleep duration is not guaranteed, which may lead to a stronger correlation between sleep duration and ICH in the elderly. In addition, we found that the association between shorter or longer night sleep duration and increased ICH scores existed only among non-drinkers. It also suggested that proper night sleep duration is better for cardiovascular health without drinking alcohol.

Taken together, sleep duration represents an important direction of cardiometabolic health risk. Future studies need to better describe the mechanisms by which sleep duration and other sleep metrics lead to morbidity. How to properly integrate sleep into seven ICH metrics and identify and refine interventions to better ameliorate this risk. At the same time, healthy sleep should be a consideration in the clinical care of patients with heart disease. Sleep may be one of the indicators used to describes the health of the cardiovascular system. Unhealthy night sleep duration is a modifiable risk factor and can provide targets for population intervention.

Our study had several strengths. Firstly, the relatively large sample size of rural population in China, as well as adjustments of a wide range of potential confounding factors, ensuring the reliability of the analysis. Secondly, to our best knowledge, few studies paid attention to the rural populations, who accounted for a large proportion of the Chinese population and had particular life styles including sleep habits. In addition, most subjects in our research are middle-aged and elderly people, which can better represent the current age structure of the Chinese rural population. Nevertheless, several limitations should also be considered. Firstly, these findings come from a cross-sectional study, rather than a prospective cohort design, thus do not accurately describe causality. Secondly, sleep duration data are self-reported and may have recall biases. However, previous studies have shown relationship between self-reported sleep duration and objective sleep duration measured by polysomnography or actigraphy (32). Therefore, the results of relatively large rural epidemiological study could reflect the prevalence of ICH in rural areas of China to some extent.

## Conclusions

There was a strong correlation between shorter night sleep duration and ICH metrics including total cholesterol, BMI and smoking, and a strong association between longer sleep and ICH metrics was observed in physical activity and blood pressure. Shorter (<6 h) and longer night (≥9 h) sleep duration were significantly associated with decreasing ICH scores and decreased odds ratio of ideal CVH. This study suggests that it may be beneficial to include night sleep duration assessment in cardiovascular risk screening. Future research should explore the causal relationship between night sleep duration and ICH.

## Data Availability Statement

The raw data supporting the conclusions of this article will be made available by the authors, without undue reservation.

## Ethics Statement

The studies involving human participants were reviewed and approved by the Zhengzhou University Life Science Ethics. The patients/participants provided their written informed consent to participate in this study.

## Author Contributions

XWu: investigation, data curation, methodology, formal analysis, visualization, and writing-original draft. XL and ZZ: investigation, data curation, writing-review, and editing. WL: investigation, validation, writing-review, and editing. SS and TA: investigation, writing-review, and editing. CW: conceptualization, methodology, investigation, validation, supervision, funding acquisition, project administration, and writing-original draft. XWa: data curation, methodology, writing-review, and editing. YL: conceptualization, methodology, writing-review, and editing. All authors contributed to the article and approved the submitted version.

## Conflict of Interest

The authors declare that the research was conducted in the absence of any commercial or financial relationships that could be construed as a potential conflict of interest.
