# Supplementary material for: Association of Night Sleep Duration and Ideal Cardiovascular Health in Rural China: The Henan Rural Cohort Study
Source: Front Public Health. 2021 Jan 11;8:606458. doi: 10.3389/fpubh.2020.606458 (PMC7830879; doi:10.3389/fpubh.2020.606458)
Supplement: Supplementary file 1 [file Table_1.DOCX]

**Supplementary Table 1. Association Between night sleep duration categories and increased IHB scores and increased IHF scores.**

| **Night sleep duration** |  | **Increased IHB scores (95%CI)** | | | |  | **Increased IHF scores (95%CI)** | | | |
| --- | --- | --- | --- | --- | --- | --- | --- | --- | --- | --- |
|  |  | **Model 1^a^** | ***p*** | **Model 2^b^** | ***p*** |  | **Model 1^a^** | ***p*** | **Model 2^b^** | ***p*** |
| **<6** |  | -0.113 (-0.145, -0.081) | <0.001 | -0.063 (-0.091, -0.035) | <0.001 |  | -0.117(-0.163, -0.071) | <0.001 | -0.056(-0.098, 0.014) | 0.009 |
| **6 to <7** |  | -0.043 (-0.064, -0.022) | <0.001 | -0.009 (-0.027, -0.009) | 0.331 |  | -0.058(-0.088, -0.028) | <0.001 | -0.031(-0.052, -0.004) | 0.025 |
| **7 to <9** |  | 1 [Reference] |  | 1 [Reference] |  |  | 1 [Reference] |  | 1 [Reference] |  |
| **≥9** |  | -0.032 (-0.058, -0.007) | 0.013 | -0.030 (-0.052, -0.008) | 0.007 |  | -0.102 (-0.138, -0.066) | <0.001 | -0.048 (-0.081, 0.015) | 0.005 |

IHB, ideal health behaviors; IHF, ideal health factors; Cl, confidence interval; OR, odds ratio. ^a^, Model 1: Unadjusted; ^b^, Model 2: Adjusted for demographic factors of age, sex, education level, income, and drinking.
